# Effectiveness of reminders to sustain practice change among direct care providers in residential care facilities: a cluster randomized controlled trial

**DOI:** 10.1186/s13012-020-01012-z

**Published:** 2020-07-01

**Authors:** Susan E. Slaughter, Misha Eliasziw, Carla Ickert, C. Allyson Jones, Carole A. Estabrooks, Adrian S. Wagg

**Affiliations:** 1 Faculty of Nursing, Edmonton Clinic Health Academy, University of Alberta, 11405 87 Avenue, Edmonton, T6G 1C9 Canada; 2 Department of Public Health and Community Medicine, Tufts University, Boston, USA; 3 Faculty of Rehabilitation Medicine, University of Alberta, Edmonton, Canada; 4 Faculty of Medicine and Dentistry, University of Alberta, Edmonton, Canada

**Keywords:** Cluster randomized controlled trial, Sustainability, Reminders, Sit-to-stand activity

## Abstract

**Background:**

The study purpose was to compare the effectiveness of monthly or quarterly peer reminder knowledge translation interventions, with monthly or quarterly paper-based reminders, to sustain a mobility innovation, the sit-to-stand activity.

**Method:**

A cluster RCT using a stratified 2 × 2 factorial design was conducted in 24 Canadian residential care facilities with 416 residents and 54 peer reminder care aides. The 1-year intervention included two intensities of reminders (high: socially based peer reminders delivered by volunteer care aides to other care aides; low: paper-based reminders posted in residents’ rooms), at two frequencies (monthly; every 3 months). Intervention fidelity was assessed using questionnaires and observations. Monthly sustainability rate of the sit-to-stand activity was calculated as the percentage of opportunities that residents successfully completed the activity in 30 days. Residents’ sustainability rates were analyzed using a linear mixed model that mirrored the clustered repeated-measures factorial trial design. The model included a random intercept to account for clustering within sites. An unstructured covariance structure characterized the interdependence of repeated measures over time.

**Results:**

Twenty-four sites were randomized. One site was excluded because of falsifying data, leaving 23 sites and 349 residents for intention-to-treat analysis. Paper reminders were implemented with high fidelity across all arms (91.5% per protocol), while the peer reminders were implemented with moderate fidelity in the monthly group (81.0% per protocol) and poor fidelity in the quarterly group (51.7% per protocol). At month 1, mean sustainability ranged from 40.7 to 47.2 per 100 opportunities, across the four intervention arms (*p* = 0.43). Mean rate of sustainability in the high intensity, high frequency group diverged after randomization, yielding statistically significant differences among the groups at 4 months which persisted for the remainder of the trial. After 12 months, the mean sustainability in the high intensity, high frequency group was approximately twice that of the other three groups combined (64.1 versus 37.8 per 100 opportunities, *p* < 0.001).

**Conclusions:**

A monthly peer reminder intervention was more effective than a quarterly peer reminder intervention, a monthly paper-based reminder intervention, and a quarterly paper-based reminder intervention, in supporting care aides to sustain a mobility innovation in residential care facilities over 1 year.

**Trial registration:**

ClinicalTrials.gov, NCT01746459. Registered 11 December 2012: https://clinicaltrials.gov/ct2/show/NCT01746459.

Contributions to the literature

- Research has shown that reminders can support the adoption of innovations in health care; however, the use of reminders to support sustainability has not been studied.
- This study provides evidence that monthly, socially based reminders can support the sustainability of an innovation for up to a year; furthermore, the reminder frequency (monthly vs quarterly) and reminder type (socially based vs paper-based) do matter.
- These findings contribute to understanding the use of knowledge translation interventions to support the sustainability of innovations in health care.

## Background

Narrowing the gap between evidence and practice is an important research focus in Canadian residential care facilities, given that the demand for these facilities is estimated to double over the next 20 years [1]. While most older Canadians remain in their homes, many will become frail during their later years and will require complex care services available in residential care facilities [1]. Working under the supervision of licensed nurses, the majority of direct care are provided by care aides (CAs), an unregulated, non-professional workforce that figures largely in the quality of care and quality of life experienced by residents in these settings [2].

Evidence to support quality of care and quality of life in residential care facilities is growing; however, little is known about best strategies to support the adoption and sustainability of evidence in practice [3]. Adoption is defined as “the intention, initial decision, or action to try or employ an innovation or evidence-based practice” [4] p.69. Sustainability is defined as the degree to which an innovation is maintained after initial efforts to ensure its adoption are complete [5]. Developing CA-targeted knowledge translation interventions is an important area of research to ensure that resources invested in ongoing education and training in residential care results in sustained practice change. In a realist review of the characteristics of practice change interventions in long-term care facilities, Caspar et al. concluded that interventions most likely to produce sustained outcomes were those with reinforcing factors to motivate continued use of new skills or practices, such as reminders, peer support, or consistent follow-up [6]. The original Promoting Action on Research Implementation in Health Services (PARIHS) conceptual framework identified three core constructs associated with implementation success [7]. In the current study, one of these core constructs, facilitation, is operationalized to be reminders as described below.

## Methods

The purpose of this clinical trial was to determine the effectiveness of a novel knowledge translation intervention, the peer reminder, compared to a standard paper reminder intervention. Specifically, the trial examined reminder intensity and reminder frequency on the 1-year sustainability of CAs completing and documenting a mobility intervention with residents living in care facilities.

### Design and participants

The protocol for this cluster randomized trial, using a stratified factorial design, is published elsewhere [8]. To summarize briefly, we recruited long-term care and assisted living facilities from the greater Edmonton region. Residents in participating facilities were invited to participate in the study if they were 65 years or older at the time of randomization, were medically stable, and were able to independently stand from a chair. Residents in participating assisted living facilities were eligible if they had been assessed by a facility case manager as meeting a minimum threshold for care requirements. Residents were recruited throughout the trial to ensure sufficient participants. CAs at participating facilities were eligible to participate as peer reminders if they were full-time or part-time employees of the facility. Executive directors or administrators of the care facilities agreed to participate in the study with the understanding that all their CA staff would be expected to support participating residents to complete the sit-to-stand activity. All participating CAs and residents (or resident proxies) provided informed written consent. The trial was approved by the Health Research Ethics Board at the University of Alberta (Pro00034781).

Between October 2013 and November 2014, CAs at all participating facilities attended standardized 20-min education sessions regarding the primary outcome, the sit-to-stand activity. The sit-to-stand activity is a functional intervention in which CAs prompted and encouraged residents to stand sit from a chair and to document residents’ responses [9, 10]. This activity has been demonstrated to slow functional decline and improve mobility in older adults [9, 11–13]. Following the completion of the majority of their education sessions, each facility underwent a minimum of a 3-month run-in phase, the adoption monitoring period, where interventions were completed to optimize adoption of the sit-to-stand activity prior to randomization [14]. As this study relied on staff documentation for the primary outcome, the adoption monitoring period helped to mitigate potential information bias by offering CAs guidance about accurate documentation.

### Reminder interventions

Two intensities of reminders (socially based peer reminders [high] or paper-based reminders [low]) were delivered at two frequencies (monthly [high] or every 3 months [low]) over a 1-year period. After consulting with site leaders, monthly and quarterly frequencies of the reminder interventions were chosen to align with the schedule of residential care documentation. The high intensity reminder, which consisted of both a socially based peer reminder and paper-based reminder, was developed by our research team for this clinical trial [15]. A peer reminder was a CA who reminded and encouraged fellow CAs to carry out a new care practice with residents. Their role was to provide brief (2–3 min) formal reminders during regularly scheduled unit meetings either once a month (high frequency), or once every three months (low frequency), depending on their randomized intervention arm [8]. The content and timing of this formal reminder was at the discretion of the peer reminder and normally lasted 5 min or less. For example, the formal reminder could be provided at the start-of-shift meeting.

Some peer reminder CAs also chose to informally provide on-unit reminders regarding completion of the sit-to-stand activity and associated documentation. Site leaders identified CAs who exhibited an interest in the sit-to-stand activity as potential candidates for the peer reminder role. Candidates had to demonstrate leadership qualities and show respect for their peers. Leadership qualities were evident when CAs demonstrated confidence and asked questions during the sit-to-stand education sessions. They may have previously assumed a leadership role such as orienting new CAs. Participation as a peer reminder was voluntary. Implementation of the peer reminder intervention was supported by intervention research assistants who met regularly with peer reminder CAs to coach them on their reminding activities. The number of peer reminder CAs in each facility varied by facility size with at least one CA peer reminder on both day and evening shifts from each unit. Drawing upon media richness theory, these peer reminders were considered high intensity because face-to-face communication with a peer providing reminders offered the possibility of delivering multiple information cues, providing rapid feedback and establishing a personal connection [16].

Low intensity, paper-based reminders were employed at all sites and were modified either monthly or once every 3 months, depending on the intervention arm. Two small (3 × 3 inch) green arrow-shaped paper reminders were placed in noticeable locations within each participating resident’s room to serve as a reminder to CAs to complete the sit-to-stand activity with the resident. The specific location was decided in collaboration with participating facilities. In addition to the arrows in the residents’ rooms, reminder posters were placed in prominent locations in nursing stations or regular meeting rooms. Visual modifications to the paper reminders, such as changing the image on the arrow, were made monthly or every 3 months to enhance their visibility; however, the green color was maintained to ensure visual recognition for staff. In contrast to high-intensity peer reminders, paper-based reminders are on the lower end of the media richness hierarchy and are theorized as low-intensity reminders. This was confirmed by site staff who were consulted on the design of the study; they noted that paper reminders are used frequently in these settings but are easy to ignore or disregard, and often blend into the setting over time.

### Randomization

Stratified randomization in blocks of four was computer-generated to ensure that sites were evenly distributed by profit-status (for-profit and not-for-profit) and residential care type (assisted living and long-term care). Allocation was concealed to ensure that the research manager was unable to influence site assignment. Investigators and research assistants collecting outcome data were blinded to the intervention assigned to the sites until all data collection was completed. Only the research manager and the intervention research assistants were aware of a site’s intervention assignment.

### Outcome measure

On a daily basis, CAs would engage a resident to complete a sit-to-stand activity, twice on the day shift and twice on the evening shift. The result of these engagements was recorded at the end of every shift on a flowsheet. Recording a successful completion of the sit-to-stand activity was considered sustainability of the activity by the CA and the resident. Recording a refusal by the resident, or the absence of notation on the flowsheet, was considered a lack of sustainability. Recording the resident as unavailable was considered missing data. Residents could contribute up to 12 months of recording depending upon their duration of participation in the trial.

### Intervention fidelity

As recommended by Slaughter et al., intervention fidelity of both the peer reminders and the paper reminders was assessed by considering dose, adherence, and participant responsiveness [17]. To assess the fidelity of the peer reminder intervention, each peer reminder completed questionnaires during the coaching meeting with the intervention research assistant. These questionnaires confirmed information on the frequency, the duration, and content of the reminder. Deviations or modifications from the protocol were noted on the questionnaires, including additional reminders either formally, informally, or in writing. At the beginning of each month, fidelity of the paper reminder intervention was assessed through direct observation to determine if the paper reminder arrows were modified or missing.

### Statistical analyses

For the purpose of analyses, the CA flowsheets were divided into consecutive 30-day periods, which were designated as months. For every 30-day period, there were up to 120 opportunities that a CA could engage a resident to complete the sit-to-stand activity. A monthly rate of sustainability was calculated for each resident as the percentage of opportunities that the resident successfully completed the sit-to-stand activity in a 30-day period. Residents’ rates of sustainability were analyzed using a linear mixed model that mirrored the clustered repeated-measures factorial trial design. A random intercept was included in the model to account for the correlation (clustering) among observations within sites, and an unstructured covariance structure (yielding the smallest Akaike’s Information Criteria (AIC) and Bayesian Information Criteria (BIC) relative to alternative covariance structures) was used to characterize the interdependence of the repeated measures over time. The saturated model consisted of eight factors: intensity (high or low), frequency (high or low), month (1 through 12), three first-order cross-products, one second-order cross-product, and the residents’ baseline rate of adoption. All statistical analyses used the intention-to-treat approach and were conducted using SAS 9.4 (SAS Institute Inc., Cary, NC). Results with *p* values less than 0.05 were considered statistically significant.

The sample size calculation, based upon a previous study, assumed an sustainability rate of 90% for the high-high arm, an sustainability rate of 55% for the single-high arms, an sustainability rate of 20% for the low-low arm, and a coefficient of variation of 0.54 among facilities to account for the clustering. A total of 24 facilities, each with an average of 15 CAs working with two residents each, yielded 80% power at a 5% 2-sided level of significance.

## Results

**Fig. 1:**
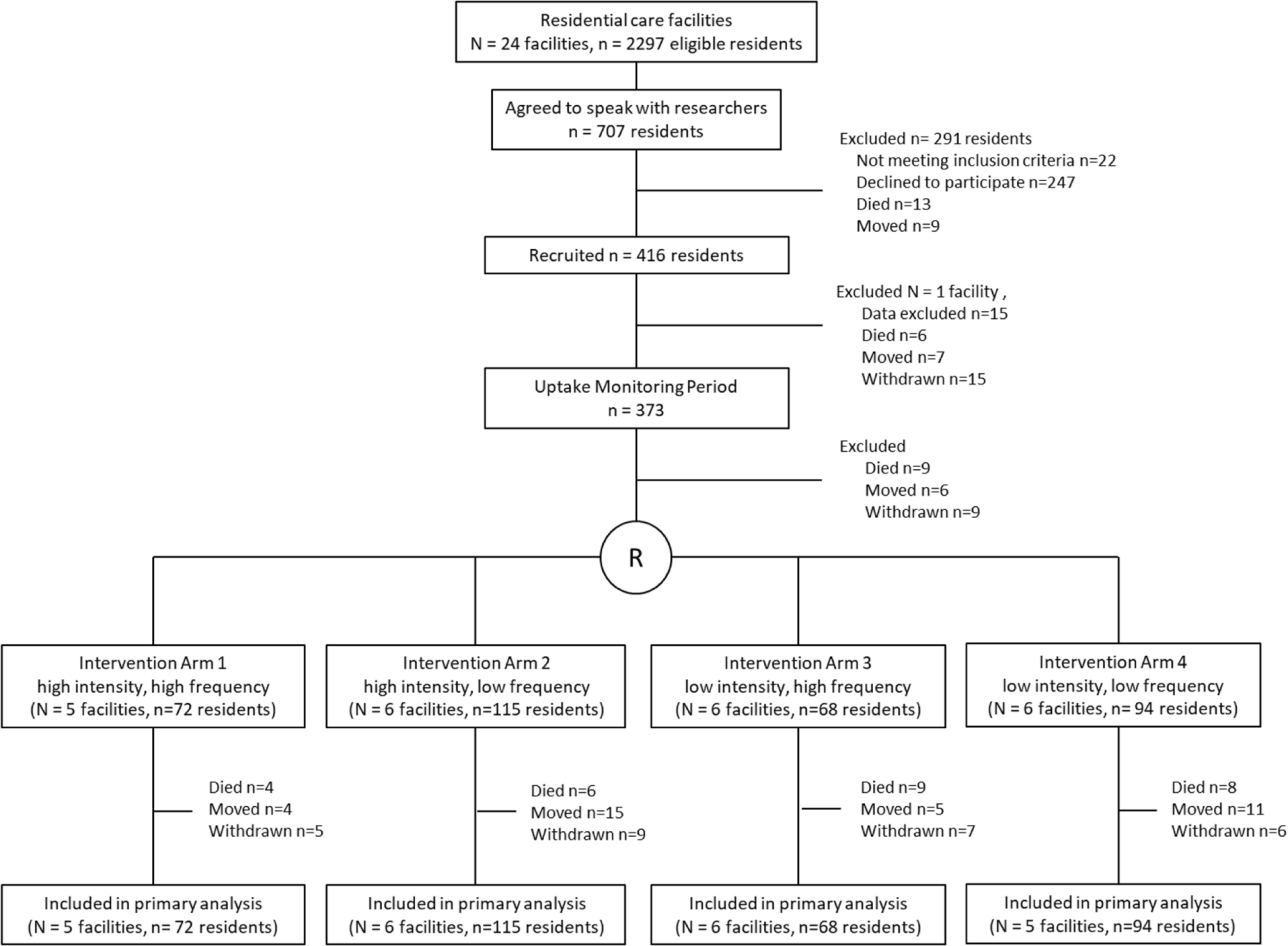
Screening, randomization, and follow-up

Sixteen supportive living and eight long-term care facilities were recruited to the study. Of the 2297 residents who met the eligibility criteria, 416 agreed to participate (Fig. 1). One facility, in which staff was falsifying data, was removed from the study. This removal combined with attrition during the run-in phase, resulted in 349 participating residents. Tables 1 and 2 summarize the characteristics of facilities and residents and peer reminder CAs, respectively. Nine CAs in the monthly sites and eight CAs in the quarterly sites were approached but declined to participate as peer reminders. The most common reason for declining was after work commitments that would conflict with the coaching sessions (*n* = 10), followed by not being comfortable with the role (*n* = 2). Eleven CA peer reminders from the monthly group and one CA peer reminder from the quarterly group dropped out of the study after completing at least one reminder and were replaced by new CA peer reminders. The most common reasons for dropping out were no longer working on the unit (*n* = 4) followed by being uncomfortable with the role (*n* = 3).

**Table 1:** Baseline characteristics of the facilities and participants

**Characteristic**	**Participants (*n*= 349)**	**High intensity, high frequency (*n*= 72)**	**High intensity, low frequency (*n*= 115)**	**Low intensity, high frequency (*n*= 68)**	**Low intensity, low frequency (*n*= 94)**
Number of sites	23	5	6	6	6
Type of site, *n* (%)					
Supportive living Long-term care	221 (63.3) 128 (36.7)	33 (45.8) 39 (54.2)	78 (67.8) 37 (32.2)	46 (67.6) 22 (32.4)	64 (68.1) 30 (31.9)
Profit status, *n* (%)					
Not-for profit For profit	188 (53.9) 161 (46.1)	38 (52.8) 34 (47.2)	64 (55.6) 51 (44.4)	40 (58.8) 28 (41.2)	46 (48.9) 48 (51.1)
Bed size					
Mean ± standard deviation	175 ± 100	226 ± 75	199 ± 137	144 ± 75	128 ± 17
Size of site, *n* (%)					
Small Medium Large	57 (16.3) 96 (27.5) 196 (56.2)	8 (11.1) 0 (0.0) 64 (88.9)	23 (20.0) 15 (13.0) 77 (67.0)	26 (38.2) 6 (8.8) 36 (53.0)	0 (0.0) 75 (79.8) 19 (20.2)
Age of resident (years)					
Mean ± standard deviation	84.0 ± 7.7	85.3 ± 7.9	84.9 ± 6.9	83.8 ± 8.6	82.0 ± 7.5
Resident sex, *n* (%)					
Female Male	235 (67.3) 114 (32.7)	47 (65.3) 25 (34.7)	86 (74.8) 29 (25.2)	44 (64.7) 24 (35.3)	58 (61.7) 36 (38.3)
Resident dementia, *n* (%)					
Yes No	230 (65.9) 119 (34.1)	43 (59.7) 29 (40.3)	76 (66.1) 39 (33.9)	44 (64.7) 24 (35.3)	67 (71.3) 27 (28.7)
Rate of sustainability (per 100 opportunities)*					
Mean ± standard error	41.0 ± 3.1	41.3 ± 7.1	43.8 ± 6.2	37.6 ± 6.6	41.0 ± 6.4

*Estimated from a linear mixed model which accounted for within-site clustering

**Table 2:** Characteristics of peer reminder care aides

**Characteristic**	**High intensity, high frequency (*n*= 33)**	**High intensity, low frequency (*n*= 21)**
Hours worked in a two-week period Mean ± standard deviation	66.4 ± 19.1	65.8 ± 15.5
Years working as a care aide Mean ± standard deviation	7.6 ± 6.5	10.0 ± 7.8
Years working on the unit Mean ± standard deviation	4.1 ± 3.5	5.10 ± 4.3
Female sex, no. (%)	29 (87.9)	19 (90.5)
Full-time employment status, no. (%)	19 (57.6)	12 (57.1)
Age, years, no. (%)		
20–29	2 (6.1)	3 (14.3)
30–39	13 (39.4)	0 (0.0)
40–49	9 (27.3)	7 (33.3)
50–59	6 (18.2)	10 (47.6)
60+	3 (9.1)	1 (4.8)
English as first language, no. (%)	12 (36.4)	7 (33.3)
High school diploma completed, no. (%)	23 (69.7)	16 (76.2)
Healthcare aide certificate completed, no. (%)	26 (81.3)	18 (85.7)
Other healthcare diploma or degree, no. (%)	7 (22.6)	7 (33.3)
Worked as RN or LPN in another country, no. (%)	9 (27.3)	4 (19.0)

*RN* registered nurse, *LPN* licensed practical nurse

In total, 263 formal reminders were provided by 33 peer reminder CAs in the monthly group, while 76 reminders were provided by 21 peer reminder CAs in the quarterly group.

The peer reminders were implemented with moderate fidelity in the monthly group (81.0% per protocol) and poor fidelity in the quarterly group (51.7% per protocol). The most common deviation from the protocol among peer reminders was providing more frequent reminders than the monthly or quarterly assignment. In the quarterly group, CAs reported formally reminding more frequently than once every 3 months: in 12 instances, they reported reminding 1–3 times per month, in 5 instances, they reported reminding weekly, and in 11, they reported reminding 5–7 times per week. Likewise, in the monthly group, CAs reported formally reminding more frequently than once every month: in 8 instances, they reported reminding 2–3 times per month, in 19, they reported reminding weekly, in 15, they reported reminding 5–7 times per week, and on 3 occasions, HCAs reported formally reminding their peers more than once per day.

The average time spent completing a formal reminder was 2.78 min (SD = 1.99; range 20 s–15 min). The average number of CAs receiving the formal reminders was 5.78 (SD 2.09; range 1–12 CAs). The average number of other staff members present (e.g., licensed practical nurses or registered nurses) was 1.84 (SD 1.07; range 0–8). Most peer reminders simply chose to verbally remind CAs to complete the sit-to-stand activity and the corresponding documentation (68.3%); however, a few chose to use a handout on various topics, praised their peers, or facilitated a discussion about success stories. The paper-based reminders were implemented with a high degree of fidelity across all intervention arms (91.5% per protocol).

**Fig. 2:**
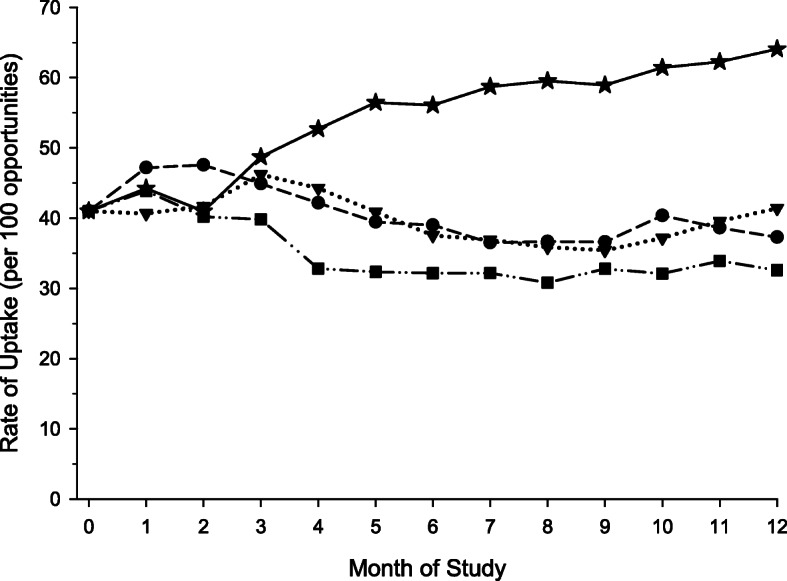
Mean rate of sustainability by month of study and intervention group**.** High intensity, high frequency (), high intensity, low frequency (); low intensity, high frequency (); low intensity, low frequency (). The mean rates of adoption were estimated from a linear mixed model that included eight factors, a random intercept, and an unstructured covariance structure. The mean rate of sustainability in the combined high intensity, high frequency group diverged shortly after randomization, yielding a statistically significant difference among the groups as early as 4 months. At the end of 12 months, the mean rate of adoption in the high intensity, high frequency group was approximately twice as high than in the other three groups combined (64.1 versus 37.8 per 100 opportunities, *p* < 0.001), which were not significantly different from each other (*p* = 0.34), and their rates of sustainability remained relatively constant over the duration of the trial

The mean rate of sustainability in the combined high intensity, high frequency group diverged shortly after randomization, yielding a statistically significant difference among the groups as early as 4 months and persisting over the remaining duration of the trial (Table 3 and Fig. 2). At the end of 12 months, the mean rate of sustainability in the high intensity, high frequency group was approximately twice as high than in the other three groups combined (64.1 versus 37.8 per 100 opportunities, *p* < 0.001), which were not significantly different from each other (*p* = 0.34), and their rates of sustainability remained relatively constant over the duration of the trial. Adding the baseline characteristics listed in Table 1, as covariates, to the linear mixed model did not alter the findings.

**Table 3:** Mean rate of sustainability (per 100 opportunities) by month of study and intervention group

	**1**	**2**	**3**	**4**	**5**	**6**	**7**	**8**	**9**	**10**	**11**	**12**
**High intensity high frequency** (95% CI)	**44.2** (37.7 *to* 50.8)	**41.0** (33.9 *to* 48.1)	**48.7** (40.9 *to* 56.5)	**52.7** (45.0 *to* 60.4)	**56.5** (48.5 *to* 64.4)	**56.1** (47.6 *to* 64.6)	**58.7** (50.5 *to* 66.9)	**59.5** (50.9 *to* 68.2)	**58.9** (50.3 *to* 67.5)	**61.4** (52.8 *to* 70.1)	**62.3** (53.4 *to* 71.1)	**64.1** (55.0 *to* 73.2)
**High intensity low frequency** (95% CI)	**47.2** (41.8 *to* 52.6)	**47.6** (41.9 *to* 53.3)	**44.9** (38.7 *to* 51.2)	**42.2** (36.0 *to* 48.4)	**39.5** (33.1 *to* 45.9)	**39.0** (32.2 *to* 45.8)	**36.5** (30.0 *to* 43.2)	**36.7** (29.7 *to* 43.6)	**36.6** (29.6 *to* 43.6)	**40.4** (33.3 *to* 47.5)	**38.6** (31.3 *to* 45.9)	**37.3** (29.8 *to* 44.8)
**Low intensity high frequency** (95% CI)	**43.9** (38.0 *to* 49.7)	**40.2** (33.8 *to* 46.6)	**39.8** (32.7 *to* 47.0)	**32.8** (25.7 *to* 40.0)	**32.4** (24.9 *to* 39.8)	**32.2** (24.2 *to* 40.1)	**32.2** (24.5 *to* 39.9)	**30.8** (22.6 *to* 39.0)	**32.8** (24.5 *to* 41.1)	**32.1** (23.6 *to* 40.7)	**33.9** (25.1 *to* 42.8)	**32.6** (23.5 *to* 41.7)
**Low intensity low frequency** (95% CI)	**40.7** (35.2 *to* 46.2)	**41.7** (35.7 *to* 47.7)	**46.2** (39.6 *to* 52.8)	**44.3** (37.8 *to* 50.8)	**40.9** (34.1 *to* 47.7)	**37.6** (30.4 *to* 44.8)	**36.9** (29.9 *to* 44.0)	**35.9** (28.4 *to* 43.3)	**35.4** (27.9 *to* 43.0)	**37.2** (29.4 *to* 44.9)	**39.6** (31.6 *to* 47.5)	**41.4** (33.2 *to* 49.6)
***P*value**^**a**^	0.43	0.30	0.39	0.003	< 0.001	< 0.001	< 0.001	< 0.001	< 0.001	< 0.001	< 0.001	< 0.001

^**a**^Comparing all four intervention groups

## Discussion

The results of the trial clearly show that only a combined high intensity, high frequency intervention was effective in significantly increasing the successful completion of the sit-to-stand activity compared to less intense and less frequent peer reminders. This suggests that monthly reminders by a coached CA can help to maintain and even improve sustainability of an intervention. To our knowledge, this is the first trial to report the effectiveness of CA peer reminders to support sustainability of an evidence-based practice. However, peer mentoring among CAs in residential care settings is not without precedent. Hegeman et al. reported the outcomes of peer mentoring in US long-term care facilities [18]. Their mentor program involved formal training of nursing assistants to develop skills in interpersonal mentoring and communication. The goal of that program was for nursing assistant mentors to work with new staff to support their integration into the care unit. The mentoring relationship, of three weeks duration, gradually tapered off once new staff were integrated into the unit. That study found higher retention rates in sites with peer mentors and demonstrated the feasibility of CA staff serving as peer mentors in long-term care; however, there was no control group in the pretest post-test study design.

Findings from our study contribute to the growing body of literature on effectiveness of knowledge translation reminder interventions. Reminders are one of the most frequently tested single knowledge translation interventions [19] with a combined median absolute improvement of care of 4.2% (interquartile range 0.8–18.8%) demonstrated in a systematic review [20]. The combination of monthly paper and socially based reminders improved sustainability by over 20%, suggesting that the social influence of peers combined with low-intensity paper reminders may be effective in producing large effects on direct care provider behavior. This is consistent with the findings of a concept analysis of knowledge transfer roles, which concluded that interpersonal contact increases the likelihood of healthcare innovations being adopted [21].

The most common deviation from the protocol was peer reminder CAs reminding their peers more frequently than their assigned intervention arm. In particular, the low fidelity in the high-intensity, low-frequency arm suggests that CAs recognized the importance of regular, frequent reminders to support sustainability of interventions in residential care. CAs demonstrated initiative and leadership by reminding their peers at more frequent intervals than requested by the intervention research assistants.

CA-led management hierarchies, where CAs take on leadership roles, are expected to become the norm in residential care facilities as reductions in professional nurse staff numbers combined with increasingly medically complex residents will necessitate modifications to traditional staffing structures [22]. CAs in Canada, particularly in long-term care facilities, are perceived as an underutilised resource by administrators [23]. Our findings suggest that CAs are capable of taking on additional roles to support the implementation of new initiatives in residential care. Future research should examine the feasibility of the peer reminder intervention to support the sustainability of other innovations in care within the CA’s scope of practice. As this study compared paper reminders alone to paper and social reminders, further study could examine the peer reminder intervention alone compared to a no reminder group, although there are ethical concerns with assigning a null control when the benefit of an intervention is evident. Evidence suggests that single interventions compared to no intervention may have larger effects than multifaceted interventions compared to single interventions [24]. Although this study used trained research educators to coach CA peer reminders, further study is required to determine if local site staff such as registered nurses or licensed practical nurses could successfully coach CA staff to work as peer reminders.

Although the major strength of this study is its cluster-randomized trial design, several limitations should be noted. We relied on documentation from the CA staff to assess sustainability of the activity. Staff may have misreported completion of the activity. Although we attempted to mitigate this risk of bias with the adoption monitoring run-in phase prior to randomization, the possibility of information bias remains. The poor fidelity of the quarterly reminder group is a limitation of the study; however, since this low fidelity was manifested by more reminders than intended in the protocol, one would expect the additional reminders to increase the sustainability of the innovation. Such was not the case. Finally, the study was conducted in a single geographic location. As such, the results may not be generalizable to residential facilities in other provinces or countries.

## Conclusions

In summary, this study demonstrated the effectiveness of a novel, peer reminder intervention to sustain a new care practice in the daily care activities of CA staff in residential care facilities. Given the increasingly important role CAs will play in the future, identifying effective knowledge translation interventions to optimize their work is an important focus for Canadian researchers. The peer reminder is a novel intervention that leverages CA resources and has the potential to support the adoption and sustainability of new care practices in this under resourced setting.

## Data Availability

The datasets supporting the conclusions of this article are available by request from the primary author (susan.slaughter@ualberta.ca).

## Abbreviations

AIC: Akaike’s Information Criteria
BIC: Bayesian Information Criteria
CA: Care aide
